# Gene knockout analysis reveals essentiality of estrogen receptor β1 (Esr2a) for female reproduction in medaka

**DOI:** 10.1038/s41598-019-45373-y

**Published:** 2019-06-20

**Authors:** Daichi Kayo, Buntaro Zempo, Soma Tomihara, Yoshitaka Oka, Shinji Kanda

**Affiliations:** 1 Department of Biological Sciences, Graduate School of Science, The University of Tokyo, 7-3-1 Hongo, Bunkyo Tokyo, 113-0033 Japan; 2 Department of Physiology, Division of Life Sciences, Faculty of Medicine, Osaka Medical College, 2-7 Daigakumachi, Takatsuki, Osaka, 569-8686 Japan; 3 Present Address: Laboratory of Physiology, Atmosphere and Ocean Research Institute, The University of Tokyo, 5-1-5 Kashiwanoha, Kashiwa Chiba, 277-8564 Japan

**Keywords:** Gene regulation, Reproductive biology

## Abstract

In vertebrates, sex steroids play crucial roles in multiple systems related to reproduction. In females, estrogens and their receptor estrogen receptor (ER or Esr) play indispensable roles in the negative sex steroid feedback regulation of pituitary gonadotropin secretion, which prevents excessive development of ovarian follicles. However, the mechanism of this feedback regulation of a gonadotropin, follicle stimulating hormone (FSH), which is essential for folliculogenesis throughout vertebrates, is poorly understood. In the present study, we generated knockouts of all subtypes of nuclear estrogen receptors in a model teleost medaka, which is suitable for the study of endocrine control and behavioral assays, and analyzed fertility, behavior and functionality of estrogen feedback in each knockout line. Among the estrogen receptors, we revealed that an estrogen receptor Esr2a plays an essential role in this feedback regulation. In addition to this, we also found that *esr2a*^−/−^ females showed oviduct atresia, which causes complete infertility. Interestingly, *esr2a*^−/−^ females showed apparently normal sexual behavior but without oviposition in response to male courtship. This phenotype indicates that physical readiness and motivation of sexual behavior is independently controlled.

## Introduction

Coordinated regulation of gonadal functions and sexual behavior by neural and endocrine systems is essential for reproduction. In female vertebrates, a group of sex steroid hormones, estrogens, which are mainly produced by ovarian granulosa cells, are suggested to play crucial roles in multiple systems^1–6^.

One of the most important roles of estrogens is the feedback regulation of gonadotropin (follicle stimulating hormone (FSH) and luteinizing hormone (LH)) secretion from the pituitary. During the folliculogenesis in female vertebrates, estrogens released from mature gonad suppress the secretion of gonadotropins from pituitary in a negative feedback manner, to prevent excessive development of ovarian follicles. In addition to such neuroendocrine regulation, estrogens are suggested to be involved in a wide variety of sex-related phenomenons, such as sexual differentiation, sexual behavior, and development of female-specific organs.

Majority of estrogenic actions are suggested to be mediated by nuclear estrogen receptors (ERs), which belong to the steroid hormone receptor family^7^. In vertebrates, two subtypes of ER, Esr1, also referred as ERα, and Esr2, also referred as ERβ^8–10^, have been generally conserved^11–13^.

In mammals, it has been strongly suggested that Esr1 expressed in hypothalamic kisspeptin neurons play essential roles in this feedback regulation of folliculogenesis by changing GnRH release from hypophysiotropic GnRH neurons and pulsatile release of LH from the pituitary^2–4,14,15^. However, growing body of evidence strongly suggests that the kisspeptin-mediated gonadotropin release can be a mammalian-specific mechanism^16–18^. Therefore, the role of estrogen receptors in the steroid feedback in mammals cannot be applied to that of vertebrates in general, and the general mechanisms of steroid feedback regulation by estrogens in vertebrates are still unknown.

A previous study has challenged to clarify the function of ERs in a model teleost zebrafish by knockout (KO) technology. However, because of their sexual plasticity, ER-KO zebrafish easily underwent sex change to males, and female functions could not be analyzed^19^. On the other hand, the sex is strictly determined by the sex determination gene, *dmy* in a teleost, medaka (*Oryzias latipes*)^20^, which led us to successfully analyze the contribution of each ER subtype to the female reproductive functions. It has been reported that teleosts possess three ERs. Esr1 and Esr2 were duplicated in the early gnathostome lineage, and Esr2 was further duplicated in the third-round whole genome duplication event in teleosts. Esr2a and Esr2b are considered to share roles of their common ancestor, Esr2, after this sub-functionalization^21–23^.

In the present study, we established ER KO lines for each ER subtype by using CRISPR/Cas9 and analyzed their phenotypes from several viewpoints such as fertility, ovarian morphology, and expression of gonadotropin genes in females of each ER KO line. From the analyses of the established line for each ER, we here provide the first experimental evidence to show that Esr2a expressed in the pituitary plays an essential role in the regulation of female reproduction, while the other two subtypes of ERs, Esr1 and Esr2b, are dispensable for that. Moreover, we also found the essential role of Esr2a in the oviduct formation, dysfunction of which results in complete infertility.

## Results

### *Esr2a*^−/−^ female medaka show complete infertility

The designed CRISPR guide RNAs successfully cleaved targeted sites of each gene and induced frameshifts for each gene. After incross and/or outcross with wild type (WT), sequence analysis was performed in the targeted genes of each ER KO medaka (Supplementary Fig. 1). We deduced the sequences of amino acid in each ER KO medaka (Supplementary Figs 2–4). The domain structures of medaka’s ERs have been predicted in the previous report^24^, and we confirmed that each ER has lost its functionality. According to the fact that all homozygotic males were fertile, we analyzed only KO females of each ER KO in the following analyses. In the present study, we used offsprings of homozygous males and heterozygous females, which includes theoretically the same number of homozygotes and heterozygotes with non-biased backgrounds in a single tank.

**Figure 1:**
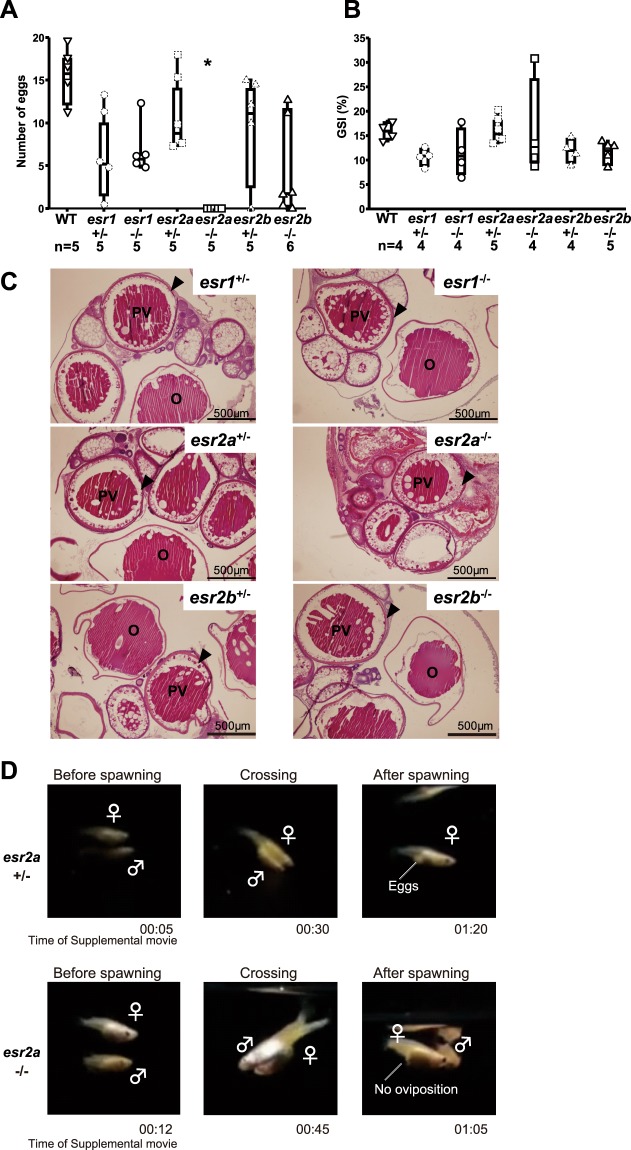
Analysis of fertility of ER KO medaka revealed complete infertility of *esr2a*^−/−^ females. (**A**) Mean number of fertilized eggs spawned by WT and ER KO females. Among ER homo knockouts, *esr2a*^−/−^ females did not show spawning (*P < 0.05, compared with WT), whereas the females of *esr1*^−/−^ and *esr2b*^−/−^ did. (**B**) Comparison of GSI between WT and each ER KO line of females. (**C**) Histological analysis of ovaries by HE staining. Females of each ER KO (age: three to four months old) showed fully developed follicles in the ovary. PV; post-vitellogenic follicle, O; ovulated egg, arrowheads; PV follicle layer composed of granulosa and theca cells. (**D**) *Esr2a*^−/−^ females showed sexual behavior without oviposition. Photos are snapshots of the movies (Supplemental Movie 1 and 2). Both *esr2a*^+/−^ and *esr2a*^−/−^ females showed sexual behavior. However, *esr2a*^−/−^ females did not show oviposition during and after the spawning acts.

We counted the number of eggs spawned by WT, hetero (*esr*^+/−^), and homo (*esr*^−/−^) KO female medaka of each ER subtype to analyze their fertility. The mean number of eggs spawned by each genotype during analyzed six days was as follows: WT, 16.0 ± 1.4; *esr1*^+/−^, 7.1 ± 2.3; *esr1*^−/−^, 7 ± 1.4; *esr2a*^+/−^, 11.6 ± 2.1; *esr2a*^−/−^, 0; *esr2b*^+/−^, 10.3 ± 2.7; *esr2b*^−/−^, 4.61 ± 2.3 (Fig. 1A, the number of eggs spawned in each day is shown in Supplementary Fig. 5). We found that *esr2a*^−/−^ female medaka were completely infertile (*P < 0.05, compared with WT), while the others could spawn more or less normally. In agreement with the description in a recent review article^25^, some of the adult females of *esr2a*^−/−^ showed abnormal bodily swelling. This abnormal swelling is probably because of the failure in oviposition due to the defective oviduct formation, which caused the accumulation of eggs.

Next, we analyzed gonadosomatic index (GSI) to assess the maturity of the ovaries. There was no significant difference among them (Fig. 1B; WT, 15.9 ± 0.89; *esr1*^+/−^, 10.6 ± 0.92, P = 0.094; *esr1*^−/−^, 11.5 ± 2.4, P = 0.5; *esr2a*^+/−^, 16.6 ± 1.3, P = 1; *esr2a*^−/−^, 16.2 ± 5, P = 0.7; *esr2b*^+/−^, 11.9 ± 1.2, P = 0.18; *esr2b*^−/−^, 11.8 ± 0.97, P = 0.12; compared with WT). For further analysis of the phenotype of each KO line, the ovaries of ER KO medaka were sectioned and were histologically examined by hematoxylin and eosin (HE) staining (Fig. 1C). As predicted by their GSI, ovaries of each ER KO line of medaka fully developed to show follicles of post-vitellogenic phase (PV). We further confirmed that there was no significant difference in the number of large preovulatory follicles (diameter > 1 mm) between *esr2a*^+/−^ and *esr2a*^−/−^ females by observing dissected ovaries (age, four to five months; *esr2a*^+/−^, 11.7 ± 1.53, n = 3; *esr2a*^−/−^, 10.3 ± 3.40, n = 4; t-test). Thus, all three subtypes of ERs were shown to be dispensable for folliculogenesis.

We next examined their sexual behavior to analyze the cause of the infertility of *esr2a*^−/−^ females. Interestingly, *esr2a*^−/−^ females showed normal acceptance behavior in response to the male courtship and showed crossing with the approaching males (Fig. 1D, n = 4, also refer Supplementary Movies 1 and 2). The courtship behavior of medaka consist of the following four steps. First, the male swims underneath the female. Second, the male swims with a characteristic movement called “quick circle” in front of the female (Supplementary Movie 1 (*esr2a*^−/−^), 00:05-00:07; Supplementary Movie 2 (*esr2a*^+/−^), immediately after the start of Movie 2). Third, the male wraps around the female’s body using male’s dorsal and anal fins (Supplementary Movie 1, 00:15-01:03; Supplementary Movie 2, 00:19-01:12), and finally, the male leaves the female (Supplementary Movie 1, 01:04; Supplementary Movie 2, 01:13). Although *esr2a*^−/−^ females showed all of those steps of normal courtship behavior, they failed to perform oviposition (Supplementary Table 2).

### Esr2a expressed in the pituitary plays a crucial role in the negative feedback regulation of *fshb* expression

**Figure 2:**
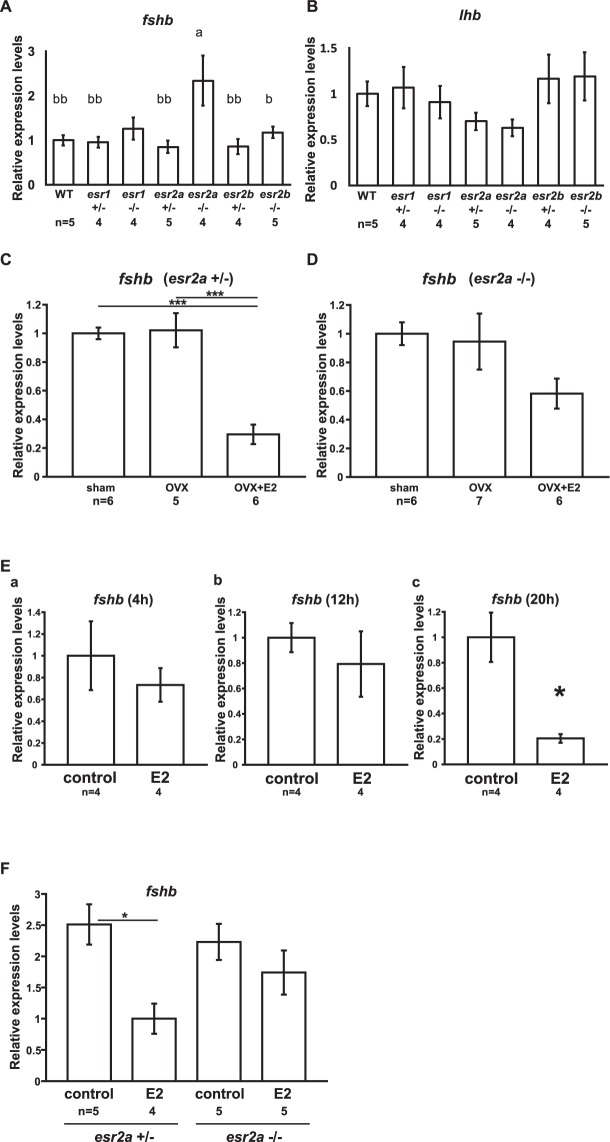
Analyses of gonadotropin expression in the pituitary showed dysfunction of *fshb* down-regulation in *esr2a*^−/−^. (**A,B**) Expression levels of *fshb* (**A**) and *lhb* (**B**) in the pituitary of each ER KO female medaka. A. The expression levels of *fshb* in *esr2a*^−/−^ females showed significantly higher than the others except *esr1*^−/−^ females (a VS b: P < 0.05, a VS bb: P < 0.01). (**B**) The expression levels of *lhb* showed no significant difference among each type of ER KO and WT females. Relative expression levels, normalized by the average expression of WT are represented in the graph (mean ± SEM). (**C,D**) Expression levels of *fshb* in the pituitary of sham, OVX, or OVX + E2 *esr2a*^+/−^ (C) and *esr2a*^−/−^ (D) female medaka. (**C**) OVX + E2 medaka of *esr2a*^+/−^ showed significant decrease in *fshb* expression compared with sham or OVX medaka (***P < 0.001). (**D**) OVX + E2 medaka of *esr2a*^−/−^ showed no significant decrease in *fshb* expression compared with sham or OVX medaka. Relative expression levels, normalized by the average expression of sham of each genotype are represented in the graph (mean ± SEM). (**E**a-c) Expression levels of *fshb* in the incubated pituitary of WT females. E2 suppressed the expression of *fshb* in the isolated pituitary after 20 hours (c) but not 4 (a) and 12 hours (b) incubation (*P < 0.05). Relative expression levels, normalized by the average expression of control group are represented in the graph (mean ± SEM). (**F**) Expression levels of *fshb* in the 20 hours incubated pituitary of *esr2a*^+/−^ and *esr2a*^−/−^ females. The pituitary of *esr2a*^+/−^ incubated with E2 showed significant decrease in *fshb* expression compared to the control of *esr2a*^+/−^ (*P < 0.05). On the other hand, inhibitory effect of E2 on *fshb* expression was diminished in the pituitary of *esr2a*^−/−^ females. Relative expression levels, normalized by the average expression of WT are represented in the graph (mean ± SEM).

To analyze gonadotropin (*fshb* and *lhb*) expression in the pituitary of KO females in each ER subtype, we performed quantitative reverse transcription PCR (qRT-PCR). *Esr2a*^−/−^ females showed significantly higher *fshb* expression than the others except *esr1*^−/−^ (Fig. 2A, a VS b; P < 0.05, a VS bb; P < 0.01). We also revealed that the expression level of *lhb* did not show significant difference among each ER KO line and WT (Fig. 2B).

Among ovarian estrogens 17β-estradiol (E2) is the most abundantly produced and shows the strongest estrogenic effect on gonadotropin expression. In the previous study using medaka that examined the effects of E2, it was reported that *fshb* expression in the pituitary is negatively regulated by E2, which suggests their involvement in folliculogenesis^26^. In the present study, because *esr2a*^−/−^ females showed higher expression levels of *fshb*, it is suggested that Esr2a mediates this E2-induced down-regulation of *fshb*. We examined reproducibility of the high expression of *fshb* in *esr2a*^−/−^ females and reconfirmed it (Supplementary Fig. 6, *P < 0.05, t- test).

To analyze the possible involvement of Esr2a in the down-regulation of *fshb* by E2, we examined E2 effects on the *fshb* expression by using ovariectomy (OVX) and OVX + E2 model in *esr2a*^+/−^ and *esr2a*^−/−^. OVX + E2 medaka of *esr2a*^+/−^ showed significant decrease in the expression of *fshb* when compared with that of sham operated (sham) or OVX medaka in a similar manner to WT^26^ (Fig. 2C, ***P < 0.001). Because of the limitation of the number of *esr2a*^−/−^ females, we could not use fully mature medaka that showed lower GSI for this experiment (Supplementary Table 3). In contrast to the results of mature females in previous study^26^, there was no significant difference between sham and OVX in *esr2a*^+/−^ females. It is probably because of the fact that intrinsic level of E2 was not high enough to trigger FSH down-regulation and thus low E2 level caused high expression in the sham-operated groups of *esr2a*^+/−^ as compared to the previous study using mature WT^26^. It is interesting to note that negative feedback regulation on *fshb* expression by exogenous E2 is functional in *esr2a*^+/−^ medaka, even when their gonads are not fully mature. On the other hand, OVX + E2 medaka of *esr2a*^−/−^ showed no significant difference in the expression level of *fshb* when compared with that of sham or OVX (Fig. 2D, P > 0.1). These results suggest that Esr2a may be involved in the down-regulation of *fshb* induced by E2.

Next, we analyzed the site of action of E2-induced down-regulation of *fshb*. To examine the possibility that the feedback regulation of E2 is mediated by Esr2a in the pituitary, we analyzed the E2 effects on the expression of *fshb* in the isolated pituitary (Fig. 2Ea–c). First, we established a protocol for short-term *in vitro* culture (incubation) of isolated pituitary. After exposure to 10 nM E2 for 20 hours (h), *fshb* expression was significantly decreased to the extent comparable to OVX + E2 treatment (Fig. 2Ea–c, *P < 0.05). We next examined the same protocol using the pituitaries of *esr2a*^+/−^ and *esr2a*^−/−^ and found that those of *esr2a*^−/−^ did not show decrease in *fshb* even in the presence of E2 (Fig. 2F). We also examined the expression of *esr2a* in the pituitary of female medaka and detected *esr2a* transcript in the pituitary (Supplementary Fig. 7). These results indicate that the negative feedback regulation on FSH is mediated by Esr2a at the pituitary level, although it does not deny the presence of some hypothalamic signals that regulate *fshb* expression.

### Esr2a females show atretic oviduct

**Figure 3:**
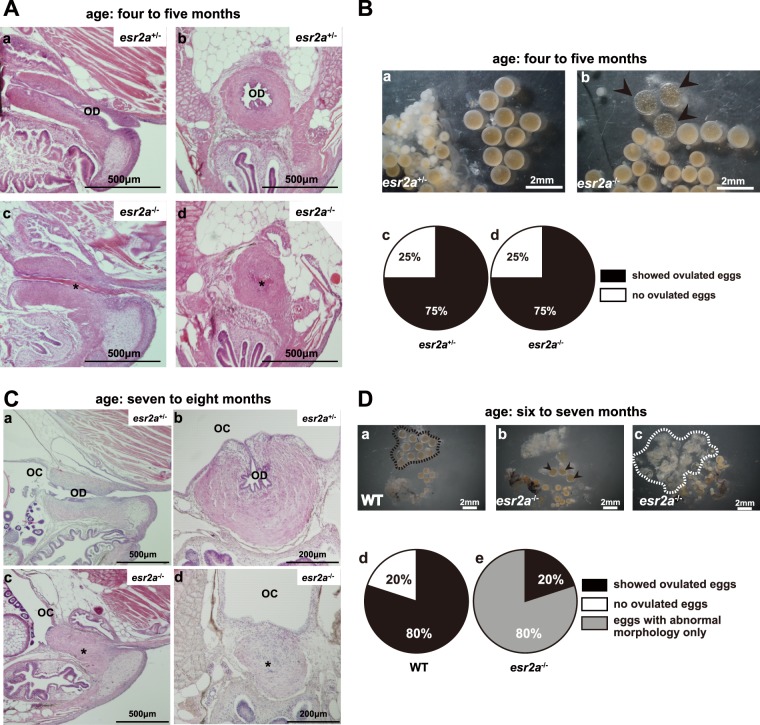
Histological analysis revealed the reason of infertility of *esr2a*^−/−^ females. (**A**) Representative sections of oviduct in *esr2a*^+/−^ (sagittal: Aa, coronal: Ab) and *esr2a*^−/−^ (sagittal: Ac, coronal: Ad) (age: four to five months). Aa, Ab. *Esr2a*^+/−^ showed oviduct with complete opening. Ac, Ad. *Esr2a*^−/−^ females showed atretic oviduct (asterisk). We examined four females of each age and genotype. Two out of four were sectioned in the sagittal plane and the others in the coronal plane. OD: oviduct, OC: ovarian cavity. (**B**) Representative photos of the ovaries in *esr2a*^+/−^ (Ba) and *esr2a*^−/−^ (Bb) (age: four to five months). Three out of four females of *esr2a*^+/−^ showed normal ovarian morphology and oviposition, which occur after the ovulation. Three out of four females of *esr2a*^−/−^ also showed normal ovulated eggs (Bb, arrow heads). Note that *esr2a*^+/−^ females showed normal ovulation and oviposition and thus they did not contain ovulated eggs in the picture “Ba”. Bc, Bd. Percentage of the females that possessed ovulated eggs with normal morphology in the ovary of *esr2a*^+/−^ (Bc) and *esr2a*^−/−^ (Bd). Black sectors indicate the percentage of females that showed ovulated eggs with normal morphology. White sectors indicate the percentage of females that showed no ovulated eggs. (**C**) Representative sections of oviduct in *esr2a*^+/−^ (sagittal: Ca, coronal: Cb) and *esr2a*^−/−^ (sagittal: Cc, coronal: Cd) (age: seven to eight months). Ca, Cb. *Esr2a*^+/−^ showed oviduct with complete opening. Cc, Cd. *Esr2a*^−/−^ females showed atretic oviduct (asterisk). Representative photos of the ovaries in WT (Da) and *esr2a*^−/−^ (Db, Dc) (age: six to seven months). Four out of five females of WT showed normal ovarian morphology and ovulated eggs (Da, enclosed by dotted black line), which occur after the ovulation. One out of five females of *esr2a*^−/−^ also showed normal ovulated eggs (Db, arrow heads) while four out of five females of *esr2a*^−/−^ showed only degenerated eggs (Dc, enclosed by dotted white line). Dd, De. Percentage of the females that possessed ovulated eggs with normal morphology in the ovary of WT (Dd) and *esr2a*^−/−^ (De). Black sectors indicate the percentage of females that showed ovulated eggs with normal morphology. Gray sectors indicate the percentage of females that showed eggs with abnormal morphology only. White sectors indicate the percentage of females that showed no ovulated eggs.

To elucidate the reason for the failure of oviposition in spite of the normal sexual behavior of *esr2a*^−/−^ females, we examined histological sections of the oviduct in *esr2a*^+/−^ and *esr2*a^−/−^ females. Adult *esr2a*^+/−^ females (age: four to five months), which spawn every day, showed oviduct with complete opening (Fig. 3Aa,Ab). In contrast, *esr2a*^−/−^ females of the same age showed atretic oviduct (Fig. 3Ac,Ad, asterisks). In these medaka, a structure that was strongly stained by eosin was observed in the oviduct (Fig. 3Ac,Ad, asterisk), which has not been shown in a previous study using wild type medaka during the development of oviduct^27^. Although it could not be determined whether this structure appears transiently in the normal development of the oviduct, we did not observe such structure at the same stage of *esr2a*^+/−^ medaka.

We analyzed the gross morphology of ovaries of randomly selected adult female medaka (age: four to five months) (Fig. 3B). The percentage of females that possessed ovulated eggs with normal morphology in *esr2a*^−/−^ at the age of four to five months were analyzed (Fig. 3Bb,Bd). For comparison, we used *esr2a*^+/−^ of the same age (Fig. 3Ba,Bc). Among the examined *esr2a*^−/−^ females, three out of four young adult females showed ovulated eggs with normal morphology (Fig. 3Bb, arrowheads; Fig. 3Bd, black sector), and only one showed anovulation (Fig. 3Bd; white sector). Although the representative picture of an *esr2a*^+/−^ ovary (Fig. 3Ba) does not contain ovulated eggs, it is considered to be because of the fact that *esr2a*^+/−^ females spawned normally to give rise to fertilized eggs. Therefore, they were judged to be able to ovulate.

These results of behavioral and histological analyses clearly indicate that the infertility of *esr2a*^−/−^ is due to the oviduct atresia. Eventually, older females (age: six to eight months) of *esr2a*^−/−^ showed complete atresia of oviduct (Fig. 3Ca–d) and degenerated eggs in their ovary (Fig. 3Da–e), while those of *esr2a*^+/−^ or WT showed normal oviduct and eggs. This phenotype suggests that ovulated eggs, which could not be oviposited because of the oviduct atresia and accumulated in the ovary, have degenerated. Interestingly, at this stage, the structure strongly stained by eosin was not observed. It is a very interesting future topic to know the identity and properties of this structure, such as the timing and the reason of its formation and disappearance.

Although the mechanism how Esr2a mediates oviduct formation or maintenance, the results obtained in the present study indicate that Esr2a is essential for the functional oviduct. We also examined the expression of *esr2a* in the oviduct of adult female medaka and detected *esr2a* transcript in the oviduct (Supplementary Fig. 7). Thus, these established *esr2a*^−/−^ female medaka may be useful for the future detailed analyses of the mechanism of the oviduct formation.

## Discussion

In the present study, we analyzed the functional roles of each ER subtype in the female reproduction by using ER KO lines of medaka generated by CRISPR/Cas9. Among them, we identified Esr2a as the mediator of estrogen feedback regulation of FSH secretion in the pituitary for the first time in vertebrates. Moreover, it was proven to be essential for oviduct formation, which is a secondary sexual characteristic of females and is critical for oviposition.

In this study, we demonstrated that Esr2a contributes to the negative feedback regulation of *fshb* expression in the medaka pituitary. The expression of *fshb* in fully mature *esr2a*^−/−^ females showed significantly increased expression (Fig. 2A and Supplementary Fig. 6). Moreover, E2 did not significantly decrease the expression level of *fshb* in *esr2a*^−/−^ OVX females (Fig. 2D), whereas E2 significantly decreased it in *esr2a*^+/−^ OVX females similar to those in the wild type^26^ (Fig. 2C). Furthermore, we also examined if E2 decrease *fshb* expression in the isolated pituitary of *esr2a*^+/−^ and *esr2a*^−/−^ females in the optimized condition (Fig. 2Ea–c) and found that E2 directly down-regulate the expression of *fshb* at the pituitary, which is mediated by Esr2a (Fig. 2F). Therefore, we here strongly suggest that Esr2a is responsible for the negative estrogen feedback on FSH secretion.

It is well known that gonadotropin negative feedback is indispensable to maintain normal reproduction in mammals. In fact, dysfunction of gonadotropin negative feedback, which results in excessive regulation of folliculogenesis, causes anovulation^28,29^. Therefore, it is possible that elevated FSH in *esr2a*^−/−^ female medaka exerted negative effects on ovarian functions. Although being a widely conserved phenomenon in vertebrates, the ER-mediated mechanism underlying the negative feedback of estrogens on FSH production remained unclear. This may be due to the concurrent suppression of FSH secretion by ovarian inhibin and estrogens in mammals^30^. Actually, in ewes, the increase in serum FSH after OVX cannot be recovered to the normal level by a single application of either E2 or inhibin, but completely recovered by simultaneous application of both^30^. On the other hand, according to the studies in teleosts, the increase in *fshb* expression after OVX was completely repressed solely by E2 application^26,31^. Taken together with present and previous studies in teleosts, it is suggested that inhibin may have relatively little effects on *fshb* down-regulation in teleosts, and *esr2a*^−/−^ medaka enabled us to demonstrate the importance of Esr2a in the negative feedback of FSH. Moreover, we revealed that E2 suppresses FSH production via Esr2a even in the absence of hypothalamus by using isolated pituitary experimental model, which strongly suggests the direct regulation at the pituitary level (Fig. 2F), although we have not elucidated whether the responsible Esr2a is localized in FSH or non-FSH cells in the pituitary. Furthermore, because of a unique situation in mammals described below, mammalian researches have concentrated on the regulation of LH, which resulted in smaller number of studies in the FSH release. However, recent studies strongly suggest that FSH is exclusively important for folliculogenesis in teleosts^32–34^ and probably other non-mammalian vertebrates as well, and thus much more attention should be paid to the regulation of FSH release for the understanding of negative feedback regulation of gonadotropin release in vertebrates. Here, we clearly indicated the mechanism of ER-mediated FSH negative feedback regulation for the first time in vertebrates by using the genome edited model organism. However, the target cells of estrogens in the pituitary is still unclear. It is therefore an interesting future topic to identify the Esr2a expressing cells in the pituitary for the further understanding of the mechanism of down-regulation of FSH production by estrogens. Future analysis using the *esr2a*^−/−^ medaka is expected to lead to the understanding of the significance of the negative feedback regulation of FSH by estrogen at the organism level, which is widely conserved in vertebrates.

In mammals, ERα (ESR1) plays crucial roles in estrogen-dependent GnRH/LH secretion because kisspeptin neurons expressing ESR1 in the arcuate nucleus (ARC) mediate negative feedback regulation by estrogens^35^. However, recent studies strongly suggest that neither kisspeptin, GnRH, nor LH is required for folliculogenesis in teleosts, and FSH is absolutely responsible for folliculogenesis^17,18,32,34,36,37^. Thus, before the emergence of mammals whose folliculogenesis is mainly regulated by kisspeptin-GnRH-LH cascade, the important site of action of negative feedback regulation of estrogens for normal folliculogenesis should be FSH secretion. Although further studies in amphibians and birds are necessary to reach conclusions, there are several evidences to support this hypothesis (for review, see^37^). In our present study, by using a model teleost medaka, we identified the most responsible ER subtype Esr2a for that. It is consistent with the present study and recent studies of ER-KO teleosts that *esr1*^−/−^ did not show any significant dysfunction in several female reproductive phenotypes thus far examined^19,38^. This finding may be widely conserved in all vertebrates except for mammals, in which kisspeptin-GnRH-LH cascade partly took over the function of FSH.

In the present study, *esr2a*^−/−^ adult females showed atretic oviduct (Fig. 3Ac,Ad,Cc,Cd). This phenotype that *esr2a*^−/−^ females produce no fertilized eggs is directly caused by this failure in oviduct formation. Previous studies using medaka suggested that estrogens are involved in the development of female-specific genital morphology^27,39^. The present study is consistent with this, and here we clearly identified the responsible ER subtype for the first time (Fig. 3, Supplementary Fig. 7). Interestingly, the ovarian cavity whose formation is also dependent on estrogens normally developed in *esr2a*^−/−^ females (Fig. 3Cc,Cd). These results suggest that Esr2a is essential for the formation of oviduct but not for that of ovarian cavity. Furthermore, in spite of this physical misformation, *esr2a*^−/−^ females displayed normal sexual behavior in response to male courtship (Fig. 1D, Supplementary Movie 1 and 2) in the presence of normally ovulated eggs in their ovary (Fig. 3Bb,Bd,Db,De). These facts strongly suggest that the neural circuits for sexual behavior are formed independently from the peripheral readiness for oviposition. The knockout models can be useful for future studies of the mechanism of sexual behavior.

**Figure 4:**
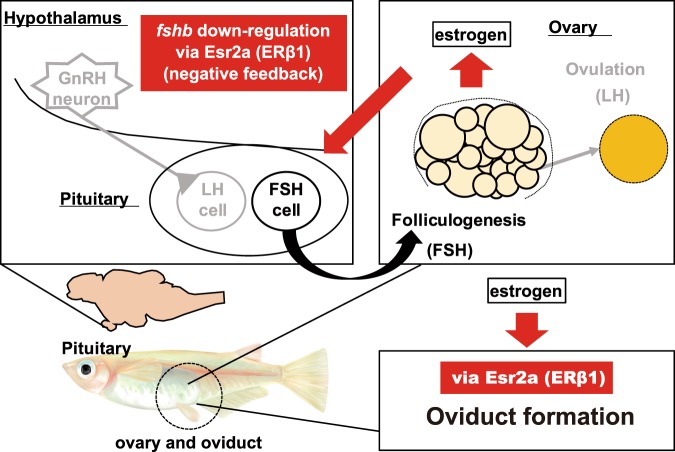
Schematic illustration showing the roles of Esr2a in reproduction in female medaka. We demonstrated that Esr2a contributes to the two important processes of reproduction; *fshb* down-regulation the formation of oviduct morphology.

In the present study, we revealed the functional contributions of ERs for reproductive regulation in female medaka. We showed that Esr2a plays essential roles in female fertility. Further analyses using *esr2a*^−/−^ females revealed, for the first time in vertebrates, the involvement of Esr2a in the FSH negative feedback regulation and the essentiality of normal oviduct morphology (Fig. 4).

In combination with the recent advancement of understanding of the central regulation of reproduction in teleosts, these ER KO lines should give us insights into the understanding of the evolution of regulatory mechanisms of reproduction in vertebrates.

## Materials and Methods

### Animals

The animals were maintained and used in accordance with guiding principle for the Use and Care of Experimental Animals of the University of Tokyo. Male and female wild-type d-rR medaka and all the ER KO medaka were maintained under a 14-hour light and 10-hour dark photoperiod (light on and off time was 08:00 to 22:00 or 09:00 to 23:00) at a water temperature of 27 °C. The fish were fed two to four times a day with live brine shrimp and flake food. All experiments were conducted in accordance with the protocols approved by the animal care and use committee of Graduate School of Science, the University of Tokyo (Permission number, 15-3).

### Generation of ER KO medaka lines by CRISPR/Cas9

We generated ER KO medaka using CRISPR/Cas9. Mixture of Cas9 mRNA, tracer RNA, CRISPR RNA, GFP mRNA diluted with PBS and 0.02% phenol red (final concentration: Cas9 mRNA, tracer RNA; 100 ng/μl, CRISPR RNA; 50 ng/μl, GFP mRNA; 5 ng/μl) was injected into the cytoplasm of one- or two-cell-stage embryos (F0). Guide RNA (gRNA) for *esr1* was transcribed by using a gRNA expression vector constructed from DR274 (Addgene, Cambridge, MA). CRISPR RNA and tracer RNA for *esr2a* or *esr2b* were synthesized by a commercial company (FASMAC, Atsugi, Japan). The injected embryos were intercrossed for the identification of the germ line founders with mutations in each target locus. Their offsprings were then outcrossed for the identification of the individual founder fish. Heterozygous transgene carriers in the F1 generation were identified by the melting curve and/or sequence analyses using primers described in the primer list (Supplementary Table 1). To obtain homozygous transgenic offspring, the carriers were crossed with each other.

### Gonadosomatic index (GSI) calculation

For the analysis of GSI of each KO line (age: three to four months), we used females that had been separated from males from the previous night of the sacrifice to avoid egg release (spawning). After confirming that they did not spawn, we dissected the ovary and measured the ovarian weight at noon. The GSI was calculated as the ovary weight/body weight × 100 (%).

To analyze the sexual maturity of females that were used in quantitative PCR (qPCR) experiment, we calculate their GSI as shown above. We used females that had not been separated from males from the previous night of the sacrifice. Thus, *esr2a*^+/−^ females that held ovulated eggs in their ovary could spawn, and their GSI must be decreased for that.

### Comparison of the number of eggs spawned

Female medaka (age: three to four months) of WT and *esr1*^+/−^, *esr1*^−/−^, *esr2a*^+/−^, *esr2a*^−/−^, *esr2b*^+/−^, *esr2b*^−/−^ were mated with WT male medaka as one to one male-female pair (Day 1). After habituation, the number of eggs spawned was counted from Day 17 to Day 22.

### Histological analysis of ovaries

Female medaka (age: three to four months) were deeply anesthetized by MS-222, and ovary samples were then treated with a Bouin’s fixative at room temperature for one to three days. After fixation, each sample was dehydrated with ethanol or methanol, cleared with xylene and embedded in paraffin. Sections of 8-9μm thickness were stained with hematoxylin and eosin (HE). The sections were observed under an upright microscope (DM5000B; Leica Microsystems, Wetzlar, Germany). Photographs were taken with a digital camera (DFC310FX; Leica Microsystems, Wetzlar, Germany).

### Analysis of sexual behavior of *esr2a*^+/−^ and *esr2a*^−/−^ females

Four to five months old female medaka of *esr2a*^+/−^ and *esr2a*^−/−^ were mated with sexually mature male medaka one by one. Movies were taken using raspberry pi and raspberry pi camera V2 (Raspberry Pi Foundation, Cambridge, UK) from ten minutes before light on to one hour after light on time, during which spawning of medaka usually occurred. We analyzed the sexual behavior for nine days.

### Analysis of gonadotropin expression in the pituitary

Female medaka of *esr2a*^+/−^ and *esr2a*^−/−^ (age: three to four months) were deeply anesthetized and their pituitaries were collected for qRT-PCR.

Total RNA was extracted from the pituitaries using the NucleoSpin RNA plus (TaKaRa, Kusatsu, Japan) according to the manufacture’s instruction. Total RNA samples were then reverse transcribed by PrimeScript^TM^ RT Master Mix (TaKaRa). For qRT-PCR, the cDNA was amplified by KAPA SYBR FAST qPCR kit (Nippon Genetics, Tokyo, Japan) with AriaMX Realtime PCR System (Agilent Technologies, Santa Clara, CA). The temperature profile of the reaction was 95 °C for 3 minutes, 40 cycles of denaturation at 95 °C for 10 seconds, annealing at 60 °C for 10 seconds, and extension at 72 °C for 10 seconds. The PCR products were verified using melting curve analysis. The data were normalized by a housekeeping gene, ribosomal protein s13 (*rps13*). The melting curve analyses were conducted to ensure that the amplicons were the same as the sequence-certified ones. The primer pairs used in the real-time PCR are listed in the primer list (Supplementary Table 1).

### Analysis of *esr2a* expression in the brain, pituitary, and oviduct

Adult female medaka of WT (age: three to four months) were deeply anesthetized and their brains, pituitaries, and oviducts were collected for reverse transcription PCR (RT-PCR) analysis. We pooled each tissue from three individuals.

Total RNA was extracted from these tissues using the FastGene^TM^ RNA Basic Kit (Nippon Genetics) according to the manufacture’s instruction. RNA samples (20 ng) were then reverse transcribed by PrimeScript^TM^ RT Master Mix (TaKaRa). For RT-PCR analysis, the cDNA was amplified by KAPA HiFi HotStart ReadyMix (Nippon Genetics) with T100^TM^ Thermal Cycler (BIO RAD, Hercules, CA). The temperature profile of the reaction was 98 °C for 1 minute, 20 cycles of denaturation at 98 °C for 10 seconds, annealing at 70 °C to 60 °C (−0.5 °C/cycle) for 5 seconds, and extension at 72 °C for 1 minute followed by second amplification step of 15 cycles of denaturation at 98 °C for 10 seconds, annealing at 60 °C for 5 seconds, and extension at 72 °C for 1 minute. The PCR products were separated on 2% agarose gels, and we verified that the amplicons matched the predicted sequence of *esr2a* cDNA by sequence analysis. The primer pairs used in the PCR are listed in the primer list (Supplementary Table 1).

### Ovariectomy and steroid treatment

Ovariectomy was performed as previously described^40^. Briefly, female medaka were anesthetized with MS222 (Sigma-Aldrich, St. Louis, MO) and their ovaries were excised via intraperitoneal operation. The OVX medaka were allowed to survive for at least four days. One group of the OVX medaka were fed with E2- (0.1 μg/individual/day) (Sigma-Aldrich) containing food in addition to the normal food for three days. After the steroid treatment, the medaka were anesthetized, and their pituitaries were collected for RT-qPCR analysis.

### Short-term incubation of isolated pituitary *in vitro*

Female medaka of WT (age: six to seven months) were deeply anesthetized and decapitated. The pituitary was collected and incubated in the medium with or without 10 nM E2 at 25 °C for 4 hours (h), 12 h, or 20 h. We prepared the medium as follow; [Leibovitz’s L-15medium (pH 7.4); catalog no. L5520; Sigma] supplemented with L-glutamine (2 mM), D-glucose (10 mM), penicillin-streptomycin (100 U of penicillin and 0.1 mg streptomycin/ml; Life Technologies, Carlsbad, CA), and 5% fetal bovine serum (JRH Biosciences, Lenexa, KS). Given the data available for previous studies^41,42^, we expect that 10 nM E2 would be a reasonable physiological concentration for a female medaka. After incubation, the pituitaries were homogenized and extracted RNA for qPCR. The total RNA extraction and qPCR were performed as described above.

Female medaka of *esr2a*^+/−^ and *esr2a*^−/−^ (age: three to four months) were deeply anesthetized and decapitated. The pituitary was collected and incubated in the medium with or without 10 nM E2 at 25 °C for 20 h. After incubation, the pituitaries were homogenized and extracted RNA for RT-qPCR as same method described above.

### Histological analysis of oviducts

Female medaka were deeply anesthetized by MS-222, and whole bodies samples were then treated with a decalcifying solution containing 10% formic acid and 70% ethanol or methanol. Then, decalcified whole bodies were dehydrated with ethanol or methanol, cleared with xylene and embedded in paraffin. Sections of 8-9μm thickness were stained with HE. The sections were observed under an upright microscope (DM5000B; Leica Microsystems). Photographs were taken with a digital camera (DFC310FX; Leica Microsystems).

### Gross anatomical examination of ovaries

WT, *esr2a*^+/−^, and *esr2a*^−/−^ female medaka were mated with WT male medaka as one or two females to one male. After habituation, we observed their ovarian morphology and took pictures with a digital camera (α-7S; SONY, Tokyo, Japan) attached to a stereoscopic microscope (SZX12; OLYMPUS, Tokyo, Japan).

### Statistics

The number of spawned eggs, and GSI of WT and ER KO were analyzed by Steel’s test, a nonparametric multiple comparison with WT control. The qPCR data were expressed as mean ± SEM. The expression levels of *fshb* or *lhb* were analyzed by Tukey-Kramer test or t-test.

## Supplementary information

Supplementary information

Supplementary movie1 esr2a homoKO

Supplementary movie2 esr2a heteroKO

## Data Availability

All data generated or analyzed during this study are included in this article and its Supplementary information files.
