# Supplementary material for: Gene knockout analysis reveals essentiality of estrogen receptor β1 (Esr2a) for female reproduction in medaka
Source: Sci Rep. 2019 Jun 20;9:8868. doi: 10.1038/s41598-019-45373-y (PMC6586646; doi:10.1038/s41598-019-45373-y)

Supplementary information

Title:

Gene knockout analysis reveals essentiality of estrogen receptor β1 (Esr2a) for female reproduction in medaka

Authors:

Daichi Kayo[1], Buntaro Zempo[1, 2], Soma Tomihara[1], Yoshitaka Oka[1], Shinji Kanda[1, 3]

[1] Department of Biological Sciences, Graduate School of Science, The University of Tokyo, 7-3-1 Hongo, Bunkyo, Tokyo 113-0033, Japan

[2] Department of Physiology, Division of Life Sciences, Faculty of Medicine, Osaka Medical College, 2-7 Daigakumachi, Takatsuki, Osaka, 569-8686, Japan

[3] Present address: Laboratory of Physiology, Atmosphere and Ocean Research Institute, The University of Tokyo, 5-1-5 Kashiwanoha, Kashiwa, Chiba, 277-8564, Japan

Supplementary Fig 1 A. Schematic diagram of targeted genes. Arrowheads indicate targeted region of CRISPR/Cas9. Each box and line indicates an exon and intron, respectively. Dotted lines indicate frameshift region. The triangles pointing to the right indicate the predicted stop codon. According to the prediction by Ensembl, Esr2a and Esr2b can be transcribed as three splice variants for each, and the representative ones are shown here. B. Alignment of genomic DNA sequences of targeted region of CRISPR/Cas9 in each ER. Red rectangles indicate deleted site of sequence: *esr1*, 7 base pair (bp) deletion (Δ7); *esr2a*, Δ2; *esr2b*, Δ17. Although most of *esr2a*$^{-/-}$ lines of medaka showed sequence described as knockout (KO) (1), a few *esr2a*$^{-/-}$ medaka showed that described as KO (2). Because all *esr2a*$^{-/-}$ females were infertile, we regarded them as the same KO line.

Supplementary Fig 2 Alignment of deduced amino acid sequences of wild type (WT) and CRISPR/Cas9 KO *esr1* gene. Arrowhead indicates the start-point of the frameshift mutation in the amino acid sequences of Esr1. DNA binding domain and ligand binding domain are indicated in red and blue shaded region, respectively. *; termination of the amino acid sequences induced by the stop codon

Supplementary Fig 3 Alignment of deduced amino acid sequences of wild type (WT) and CRISPR/Cas9 KO *esr2a* gene. Arrowhead indicates the start-point of the frameshift mutation in the amino acid sequences of Esr2a. DNA binding domain and Ligand binding domain are indicated in red and blue shaded region, respectively. *; termination of the amino acid sequences induced by the stop codon

Supplementary Fig 4 Alignment of deduced amino acid sequences of wild type (WT) and CRISPR/Cas9 KO *esr2b* gene. Arrowhead indicates the start-point of the frameshift mutation in the amino acid sequences of Esr2b. DNA binding domain and Ligand binding domain are indicated in red and blue shaded region, respectively. *; termination of the amino acid sequences induced by the stop codon

Supplementary Fig 5 The number of eggs spawned in the Day 17-22. WT (A), *esr1*$^{+/-}$ (B), *esr1*$^{-/-}$ (C), *esr2a*$^{+/-}$ (D), *esr2b*$^{+/-}$ (F), *esr2b*$^{-/-}$ (G). Inverted triangle and whisker indicate the mean number of eggs spawned and ± SEM, respectively.

Supplementary Fig 6 Expression levels of *fshb* in the pituitary of *esr2a*$^{+/-}$ and *esr2a*$^{-/-}$ females (*: $P<0.05$). We reconfirmed the reproducibility of the high expression of *fshb* in *esr2a*$^{-/-}$ females. Relative expression levels, normalized by the average expression of *esr2a*$^{+/-}$ are represented in the graph (mean ± SEM).

Supplementary Fig 7 RT-PCR analysis of *esr2a* from the pituitary and oviduct of adult female medaka. We amplified *esr2a* transcript (2100bp) including the whole predicted open reading frame. RT (+) and RT (-) PCR templates were subjected to the thermal profile of reverse transcription with or without transcriptase, respectively; M, DNA ladder marker. The picture displayed below is full-length of the gel. DNA ladder marker indicate 250bp, 500bp, 750bp, 1000bp (increased intensity), 1500bp, 2000bp, 2500bp, 3000bp

(increased intensity), 4000-10000bp (later bands) length of DNA.

Supplementary Table 1

Primer name	Gene	Primer sequence (5'-3')	Ensembl gene ID
**Sequence**			
era ex2 seq se2	*esr1*	AGGGACGTACGACTATGCCG	ENSORLG00000014514
era ex2 seq as2	*esr1*	GTGGCTGGGTGGATGCATAA	
erb1 ex2 seq se2	*esr2a*	TCCCAGGAGAGTGAAGAGGC	ENSORLG00000017721
erb1 ex2 seq as2	*esr2a*	CCTCTTGAAGAAGGCCTTGC	
b2 pcr se1	*esr2b*	CCTCCCCTGGGCTGAATAATG	ENSORLG00000018012
b2 pcr as1	*esr2b*	GAGGAGGGACTGTCAGTCAG	
**qRT-PCR**			
rps13 F	ribosomal protein subunit 13 (*rps13*)	GTGTTCCCACTTGGCTCAAGC	ENSORLG00000001289
rps13 R	*rps13* qRT-PCR	CACCAATTTGAGAGGGAGTGAGAC	
qPCR LHb F	*lhb* qRT-PCR	TGCCTTACCAAGGACCCCTTGATG	ENSORLG00000003553
qPCR LHb R	*lhb* qRT-PCR	AGGGTATGTGACTGACGGATCCAC	
qPCR FSHb F	*fshb* qRT-PCR	TGGAGATCTACAGGCGTCGGTAC	ENSORLG00000029237
qPCR FSHb R	*fshb* qRT-PCR	AGCTCTCCACAGGGATGCTG	
**RT-PCR**			
esr2a se3	*esr2a*	CTGTGCCTCAGAACCGTGGATCTTA	ENSORLG00000017721
esr2a as4	*esr2a*	GTTTAGCTGAGACGAGTGTCGAAC	

Supplementary Table 2

	showed sexual behavior	showed spawning
esr2a$^{+/-}$	4/4	4/4
esr2a$^{-/-}$	4/4	0/4

Supplementary Table 3

		GSI
Fig. 2A	***esr2a***$^{+/-}$	**16.6 ± 1.3**
	esr2a$^{-/-}$	**16.2 ± 5.0**
Fig. 2C	***esr2a***$^{+/-}$	**3.2 ± 0.4**
	esr2a$^{-/-}$	**5.0 ± 1.1**
Supplementary Fig. 6	***esr2a***$^{+/-}$	**9.4 ± 0.5**
	esr2a$^{-/-}$	**14.3 ± 0.8**

Supplementary Movie 1. Representative movie of sexual behavior of an *esr2a*$^{-/-}$ female for two minutes including spawning behavior.

Esr2a$^{-/-}$ female accepted male courtship (time, 00:15-01:03) but failed to oviposit.

Supplementary Movie 2. Representative movie of sexual behavior of an *esr2a*$^{+/-}$ female for two minutes including spawning behavior.

The *esr2a*$^{+/-}$ female accepted male courtship (time, 00:19-01:12) and showed normal oviposition.

Supplemental figure 1

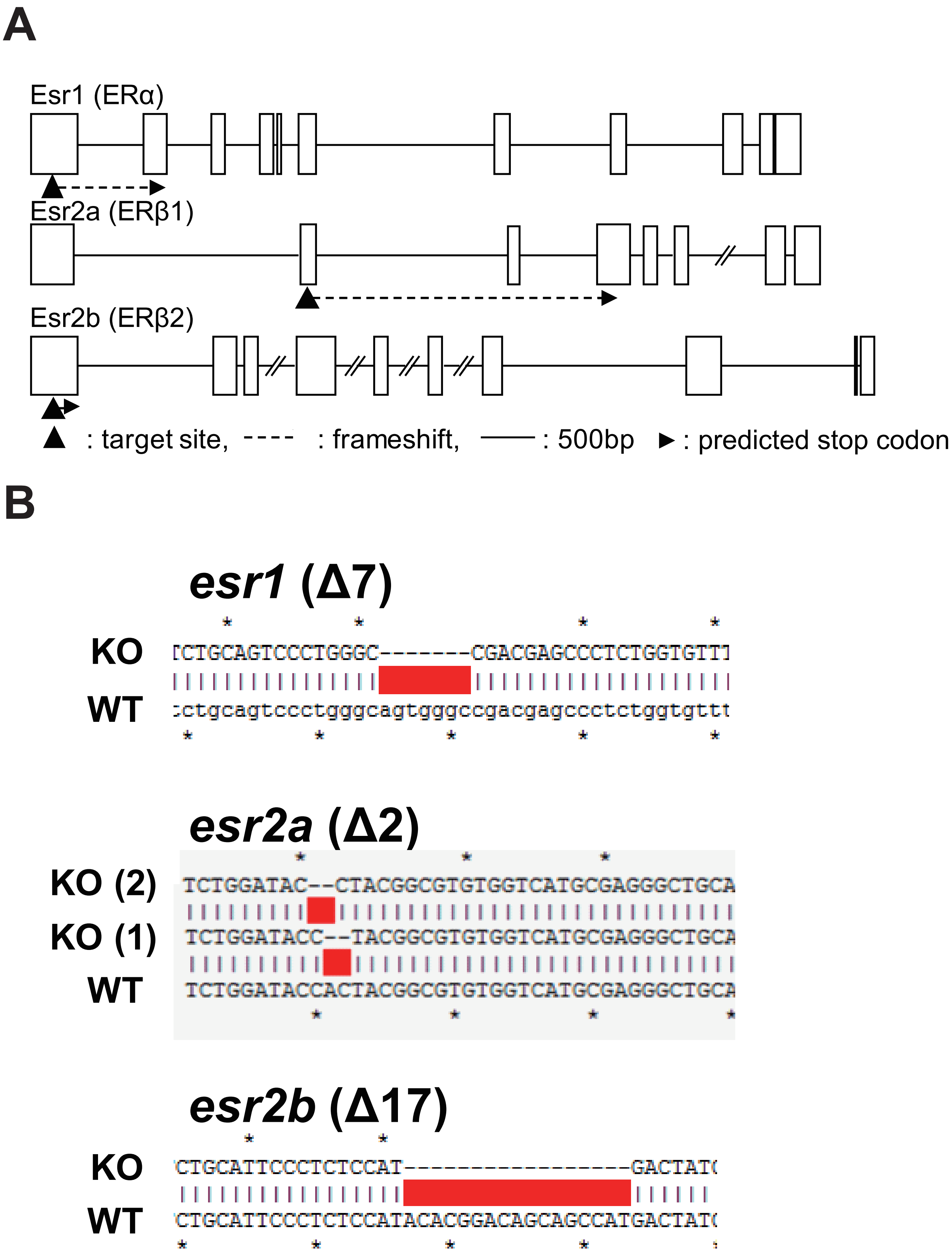

Supplemental figure 2

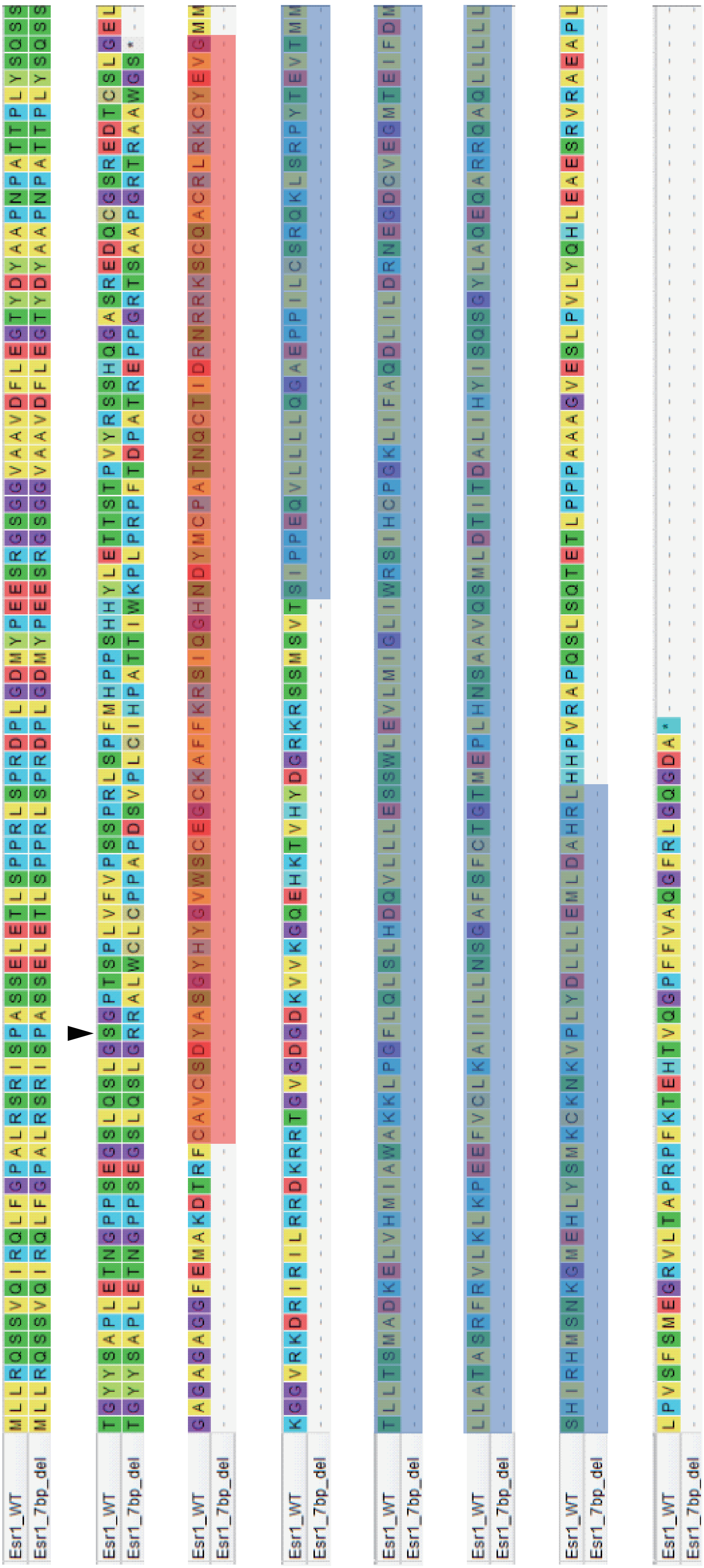

Supplemental figure 3

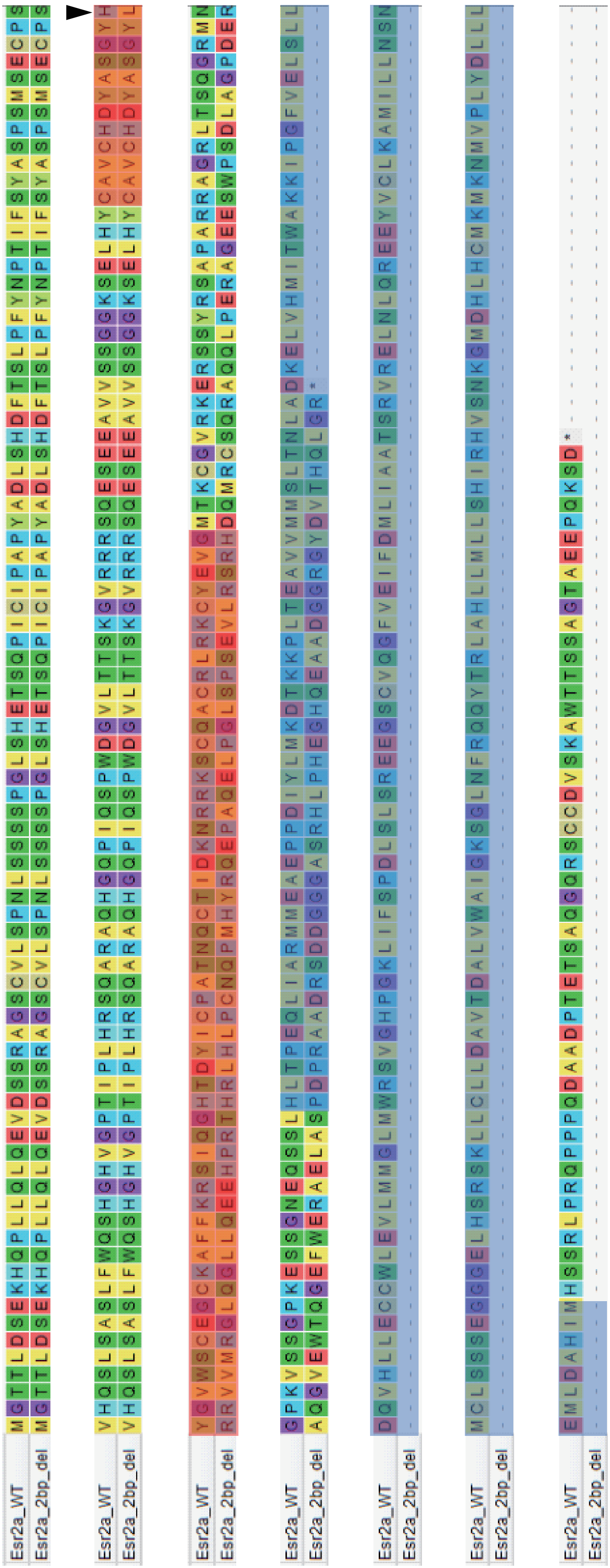

Supplemental figure 4

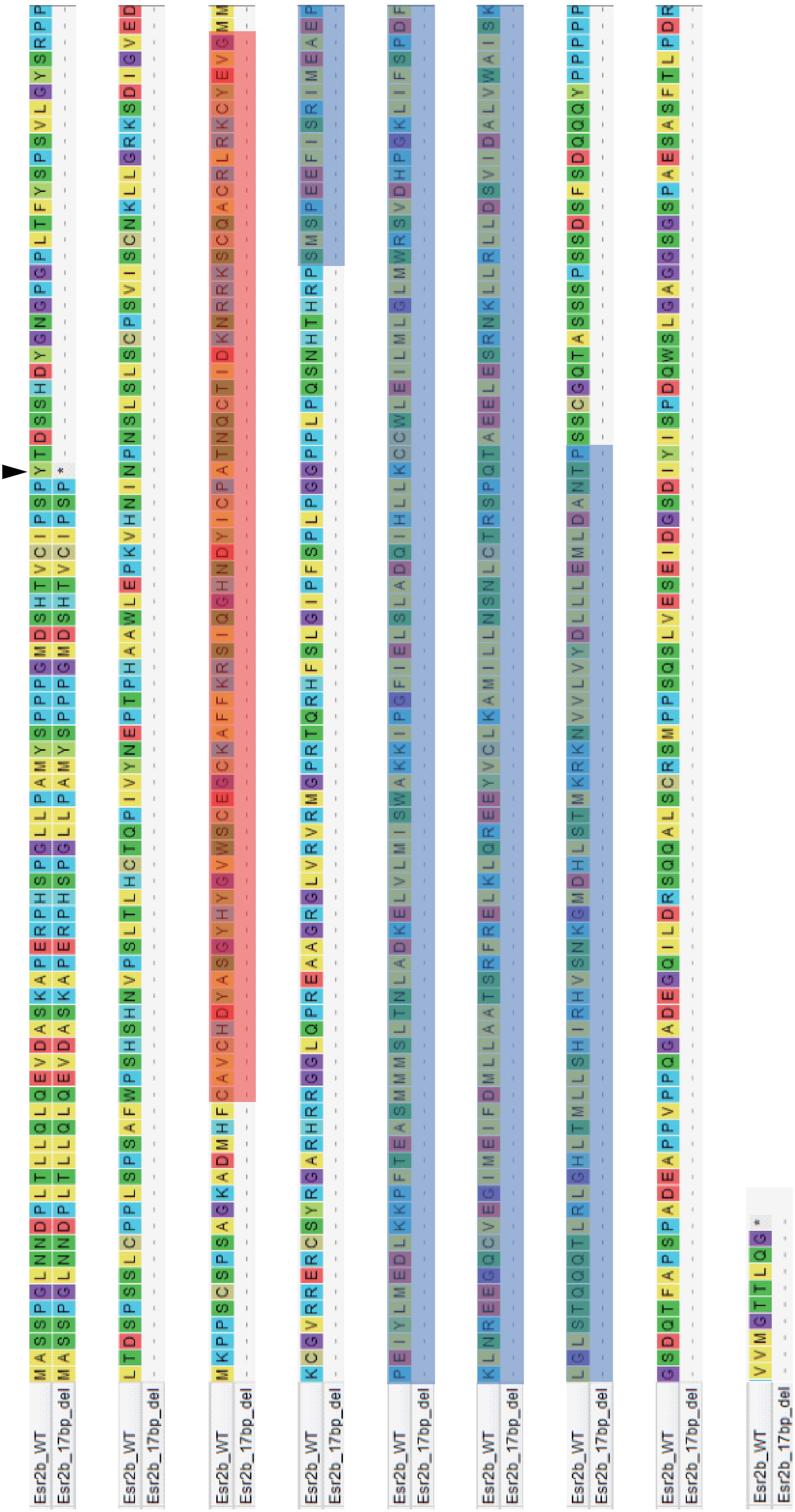

Supplemental figure 5

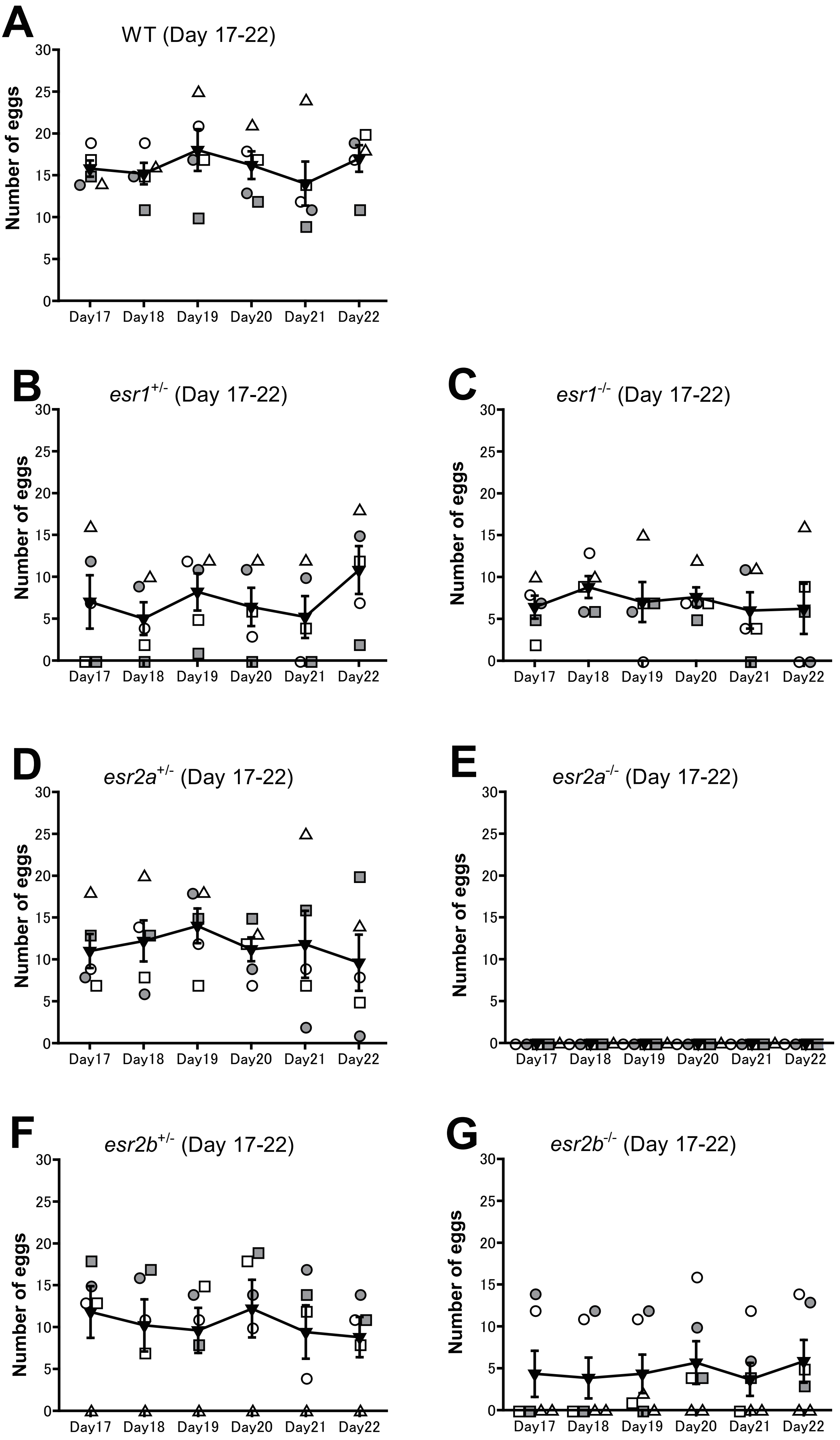

Supplemental figure 6

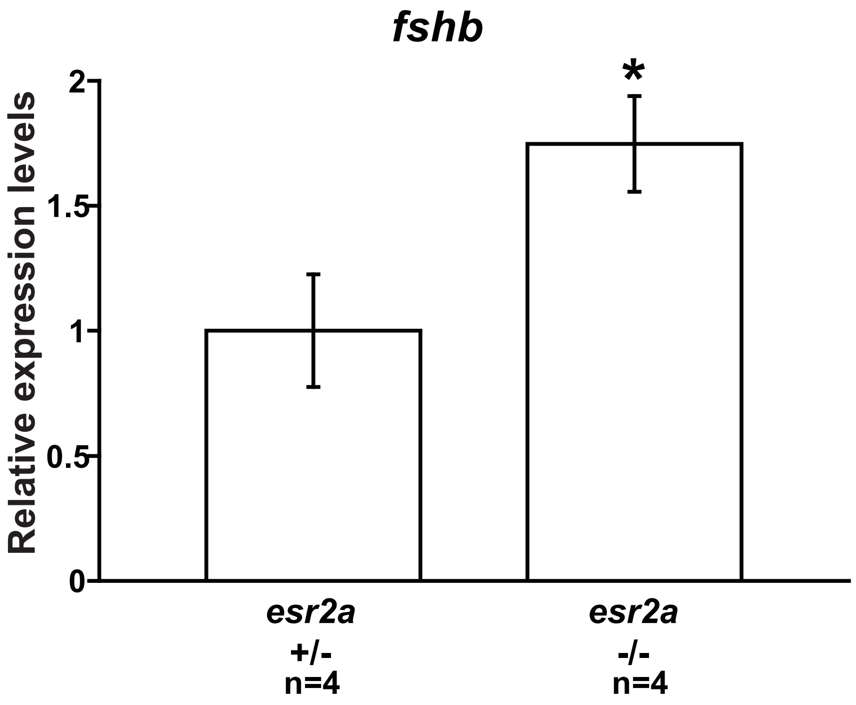

Supplemental figure 7

esr2a

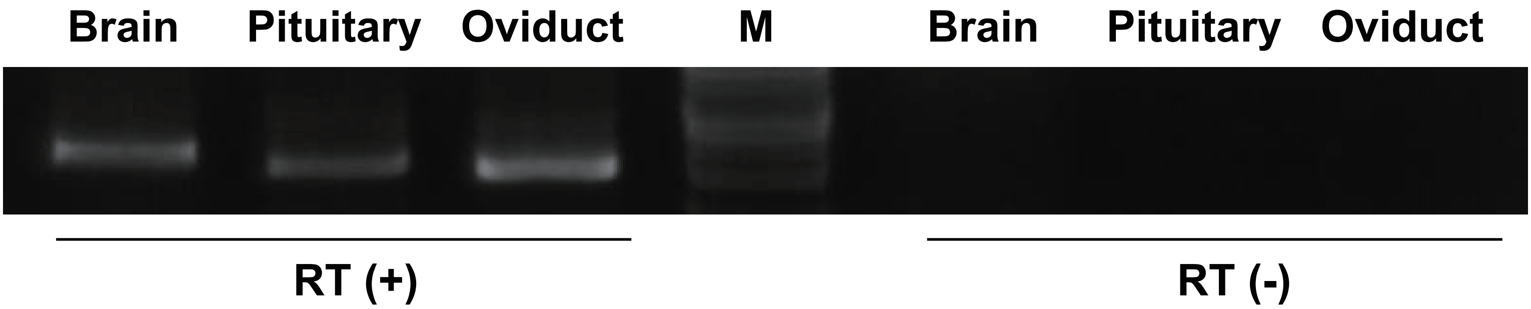

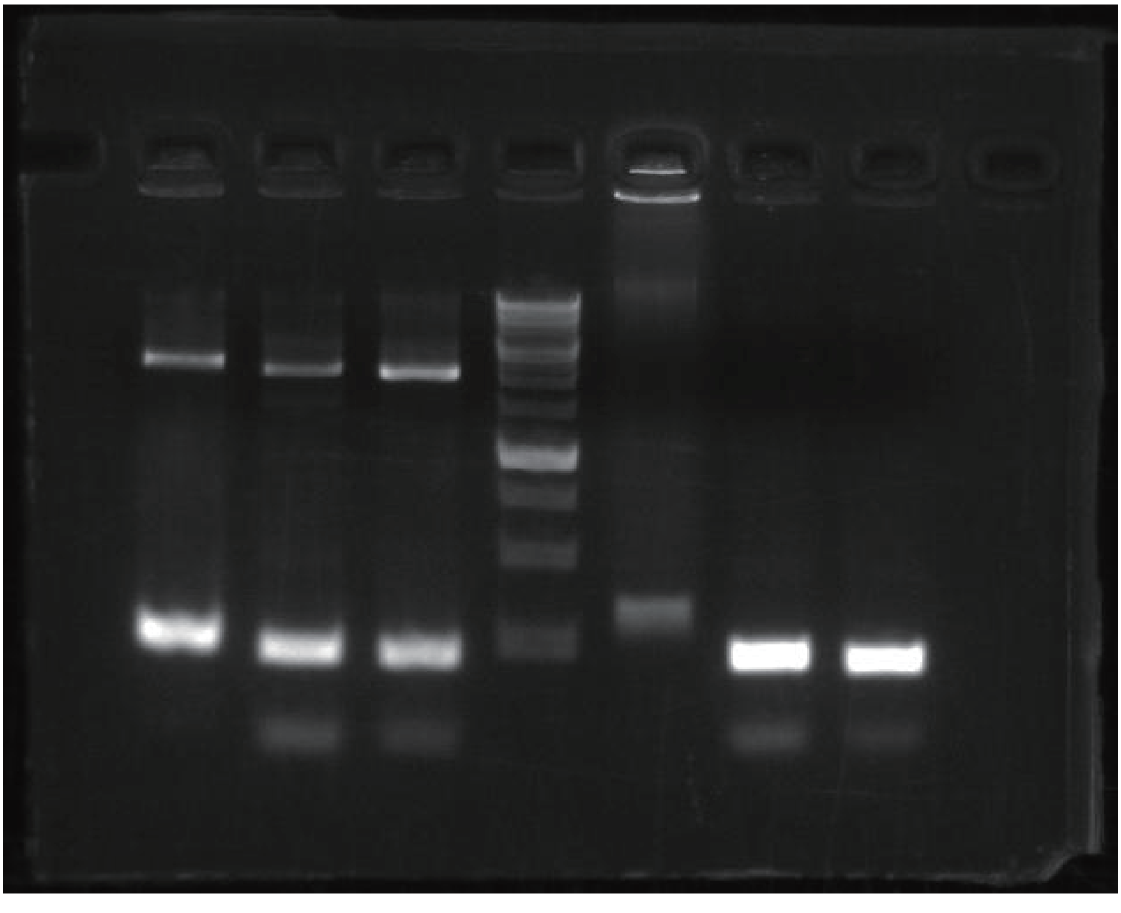

Supplement: Supplementary file 1 — Supplementary information [file 41598_2019_45373_MOESM1_ESM.pdf]
